# Cannabis lighting: Decreasing blue photon fraction increases yield but efficacy is more important for cost effective production of cannabinoids

**DOI:** 10.1371/journal.pone.0248988

**Published:** 2021-03-23

**Authors:** F. Mitchell Westmoreland, Paul Kusuma, Bruce Bugbee

**Affiliations:** Crop Physiology Laboratory, Utah State University, Logan, Utah, United States of America; National Research Council of Italy, ITALY

## Abstract

LED technology facilitates a range of spectral quality, which can be used to optimize photosynthesis, plant shape and secondary metabolism. We conducted three studies to investigate the effect of blue photon fraction on yield and quality of medical hemp. Conditions were varied among studies to evaluate potential interactions with environment, but all environmental conditions other than the blue photon fraction were maintained constant among the five-chambers in each study. The photosynthetic photon flux density (PPFD, 400 to 700 nm) was rigorously maintained at the set point among treatments in each study by raising the fixtures. The lowest fraction of blue photons was 4% from HPS, and increased to 9.8, 10.4, 16, and 20% from LEDs. There was a consistent, linear, 12% decrease in yield in each study as the fraction of blue photons increased from 4 to 20%. Dry flower yield ranged from 500 to 750 g m^-2^. This resulted in a photon conversion efficacy of 0.22 to 0.36 grams dry flower mass yield per mole of photons. Yield was higher at a PPFD of 900 than at 750 μmol m^-2^ s^-1^. There was no effect of spectral quality on CBD or THC concentration. CBD and THC were 8% and 0.3% at harvest in trials one and two, and 12% and 0.5% in trial three. The CBD/THC ratio was about 25 to 1 in all treatments and studies. The efficacy of the fixtures ranged from 1.7 (HPS) to 2.5 μmol per joule (white+red LED). Yield under the white+red LED fixture (10.4% blue) was 4.6% lower than the HPS on a per unit area basis, but was 27% higher on a per dollar of electricity basis. These findings suggest that fixture efficacy and initial cost of the fixture are more important for return on investment than spectral distribution at high photon flux.

## Introduction

*Cannabis* is a high value crop that can be profitably grown in controlled environments under sole-source electric lights [1], but the cost of electricity is a high fraction of overall production costs [2]. High-pressure sodium (HPS) lights are commonly used in *Cannabis* cultivation because they have a low upfront cost and high photon output. However, advances in light-emitting diode (LED) technology [3, 4] has led to more efficient fixtures (watts of output per watt of input), but these fixtures vary in their efficacy (micromoles of photons per joule of energy input; μmol J^-1^), depending on the choice of LED and drive current [5]. These differences in efficacy have a significant impact on energy use in controlled environment plant production. LED fixtures can be made with unique spectra that have the potential to increase flower yield and quality (cannabinoid profile) [6–8].

Spectral effects on photosynthesis have been studied for over 70 years (Hoover 1937). McCree [9, 10] and Inada [11] found that the quantum yield (moles of carbon fixed per mole of absorbed photon) of red (601 to 700 nm) photons was about 25% greater than blue (401 to 500 nm) photons and about 5% greater than green photons (501 to 600 nm). Yield photon flux (YPF) considers this photosynthetic response and weights photons from 300 to 800 nm according to their relative quantum yield [12, 13]. Although this spectral effect on quantum yield is well known, YPF has not been widely used to define photosynthetic photons. Instead, the widely used standard is the photosynthetic photon flux density (PPFD), defined as the number of photons within the waveband of photosynthetically active radiation (PAR; 400 to 700 nm) per square meter and second. PPFD assumes all photons in this range equally drive photosynthesis. Although this is not strictly correct, McCree [10] applied the equal weighting standard to the commercially available lighting technologies at the time (before LEDs were available) and concluded that this simpler PPFD definition adequately predicted quantum yield. At the time, spectroradiometers were slow, heavy, non-portable, and expensive, and the equal weighting definition could be used to make a lower cost quantum sensor.

The traditional definition of PAR includes photons between 400 and 700 nm, but recent studies now indicate that far-red photons (701 to 750 nm) are equally efficient at driving photosynthesis when coupled with shorter wavelengths [14, 15]. Far-red photons preferentially excite photosystem I in the photosynthetic electron transport chain, effectively releasing a bottleneck on the pool of reduced plastoquinone and photosystem II, thus allowing for a more rapid re-oxidation and more efficient photosynthesis [16].

As demonstrated by studies under monochromatic spectra or low PPFD—blue, green and red photons can alter plant morphology. Numerous studies have shown no effect of substituting green and red photons under a constant fraction of blue photons. For example, Son and Oh [17] and Kang et al. [18] demonstrated that substituting 10% red photons with green photons had no effect on leaf area or plant diameter in lettuce under 10, 20 or 30% blue. Snowden et al. [19] saw little to no effect on morphology in multiple species when increasing the fraction of green from 2 to 41% at about 11% blue. By contrast, multiple studies (including the studies that found no effect of substituting green and red photons) have shown a reduction in leaf area and dry mass gain with an increasing blue photon fraction from 5 to 30% [17–25].

Most spectral studies have investigated responses to blue photons at a PPFD of 500 μmol m^-2^ s^-1^ or less, but blue photon responses can interact with intensity [19, 21], so it is difficult to extrapolate the findings on blue photon response at low PPFD to intensities greater than 500 μmol m^-2^ s^-1^. This is particularly important with *Cannabis*, which has an increasing rate of photosynthesis up to a PPFD of 1500 μmol m^-2^ s^-1^ [26] and is increasingly being grown under high PPFD in commercial cultivation.

Far-red photons can also alter plant morphology by increasing stem elongation and leaf expansion, which typically increases radiation capture and thus yield [25, 27, 28]. The mechanism underlying these effects is well studied. Activation of the photoreceptor phytochrome (Pfr) inhibits the activity of a group of transcription factors called PHYTOCHROME INTERACTING FACTORS (PIFs). PIFs promote the expression of genes related to elongation, including auxin synthesis. Increased far-red fraction inactivates the phytochrome and releases the inhibition of PIFs, which promotes auxins and thus increases cell elongation [29].

Cannabinoids are a unique class of secondary metabolites that are synthesized by the genus *Cannabis*. Since Gaoni and Mechoulam [30, 31] first described the structure and synthesized tetrahydrocannabinol (THC) and cannabidiol (CBD) *in vitro*, more than 150 cannabinoids have been described [32, 33]. Cannabinoid synthesis occurs primarily in capitate-stalked glandular trichomes that are highly concentrated on the bracts of pistillate flowers [34]. The biosynthetic pathway *in vivo* has been extensively studied and is primarily under genetic control [32, 35–37], although environmental factors can influence the final cannabinoid concentrations [38]. Environmental effects on cannabinoid concentration are of significant interest as the distinction between marijuana (greater than 0.3% THC) and hemp (less than 0.3% THC) has become a legal concern and the demand increases for medical grade *Cannabis* with predictable and consistent cannabinoid profiles [39–41]. The beneficial effects of spectral quality on secondary metabolism has been well studied [42, 43], so the use of unique spectra to regulate cannabinoid biosynthesis in controlled environment *Cannabis* production warrants exploration.

Early work on *Cannabis* suggested that PPFD [44, 45], spectral quality [46], and photoperiod [47] can alter cannabinoid biosynthesis. Mahlberg and Hemphill [46] used Plexiglas filters to provide monochromatic light to greenhouse grown hemp and found that leaves of plants grown under a red filter contained 3 times higher cannabinoid concentration than plants grown under blue and green filters. They also reported a shift in the ratio of cannabinoids, in particular cannabichromene (CBC) and THC, under monochromatic filters compared to sunlight. However, there were differences in non-photosynthetic radiation among treatments, which make the results difficult to interpret. Nonetheless, this early study provided evidence that radiation changes can influence cannabinoid concentration.

LED technology has facilitated a more rigorous investigation into the effect of spectral quality on cannabinoid production [7]. Magagnini et al. [6] reported significant increases in flower yield among plants of a high THC *Cannabis* variety grown under mogul-base HPS (8% blue) compared to two LED fixtures with 14% and 24% blue, but plants grown under HPS had a lower cannabinoid concentration than the two LED treatments. Notably, the total amount of cannabinoids (cannabinoid yield) was not significantly different among the treatments. Namdar et al. [8] found significant differences in morphology and cannabinoid concentration among plants grown under LEDs, T5 fluorescent tubes, and HPS at different growth stages, but the PPFD ranged from 90 to 500 μmol m^-2^ s^-1^ among the three spectral treatments, making these results difficult to interpret. A PPFD of 90 μmol m^-2^ s^-1^ is only slightly above the light compensation point [26]. The results of Namdar [8] are likely caused by differences in photon flux rather than spectral quality, as intensity typically has a large impact on growth [1, 48].

Our objectives were twofold: 1) investigate the effect of spectral quality at a constant high PPFD on yield and quality of *Cannabis* in a controlled environment and 2) evaluate the effect of fixture efficacy on economic yield. We hypothesized that yield would increase as the fraction of blue photons decrease, but fixture efficacy would have a larger impact on the economics of indoor *Cannabis* cultivation.

## Materials and methods

### Environmental conditions

**Fig 1:**
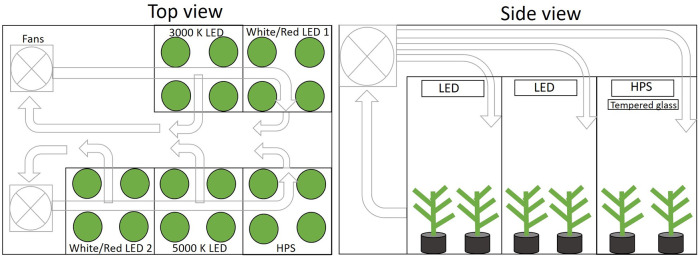
A schematic diagram of the walk-in growth room with five, photon-independent chambers and common atmospheric conditions.

It is critical to maintain constant environmental conditions other than the variable being studied. To achieve constant conditions among treatments within a study, plants were grown in a walk-in growth room that had five 1 m^2^ photon-independent sections and common atmospheric conditions (Fig 1).

Each independent section had white reflective walls to precisely define the growth area and simulate the presence of additional plants around the perimeter. Went [49] pioneered this approach at the CalTech Phytotron. Reflective side-walls are commonly used to minimize guard row effects and facilitate extrapolation to larger areas. They have been used in multiple studies of canopy photosynthesis [15, 50, 51]. In all studies, the canopy filled the entire chamber by about week 3.

Overhead fans provided continuous, ample air exchange of 0.8 meters per second at the top of the canopy, measured with a hot-wire anemometer (TSI, Inc. model 8330). The room was uniformly enriched to 1000 ± 50 ppm CO_2_, continuously measured with an infra-red gas analyzer (LI-COR, Inc., model 850). Temperature was measured with a shielded, fan-aspirated, precision thermistor (Apogee Instruments Inc., model ST-100) mounted at canopy height in each chamber. Humidity was measured with a temperature and relative humidity sensor (Campbell Scientific Inc., model HMP45A).

Measurements were made every 10 seconds and five minute averages of all environmental data were recorded with a data logger (Campbell Scientific Inc., model CR1000). Fixtures were randomized among chambers in each trial. The environmental conditions of each trial are listed in Table 1.

**Table 1:** Environmental conditions for three trials.

Trial	PPFD	Day temperature (°C)	Night temperature (°C)	Relative humidity (%)	Vapor pressure deficit (kPa)
1	750 ± 30	21.6 ± 0.5	17.1 ± 0.6	54.1 ± 15.8	1.4 ± 0.8
2	900 ± 30	24.7 ± 0.7	21.3 ± 1.1	57.9 ±2.6	1.3 ± 0.1
3	900 ± 30	26.5 ± 0.5	25.6 ± 0.9	63.7 ± 6.9	1.2 ± 0.3

Values are mean ± standard deviation over time. Trial two was harvested one week early due to a decline in cannabinoids observed at week six.

### Spectral treatments

Spectral treatments included a double-ended high-pressure sodium fixture (DE-HPS), a warm white LED fixture (3000 K), a cool white LED fixture (5000 K) and two white+red combination LED fixtures (white+red 1 & white+red 2). These combinations were selected to achieve a relatively constant fraction of green and red photons while providing a five-fold difference in blue photons (Table 2). These fixtures represent an industry standard fixture (HPS) and a low to high range of efficacies for LED fixtures [52]. The efficacy for DE-HPS was the average of the flat plane integration method and integrating sphere method described in Nelson and Bugbee [3]. The remaining fixtures were independently tested by TÜV SÜD America.

**Table 2:** Efficacy, spectral distribution, and incoming radiation of the five spectral treatments.

	DE-HPS	3000 K	White + Red 1	White + Red 2	5000 K
**Efficacy (μmol J**^**-1**^**)**	1.72	2.13	2.51	2.40	2.43
**% Blue (400–500 nm)**	4	10	10	16	20
**% Green (501–600 nm)**	43	39	41	40	49
**% Red (601–700 nm)**	53	51	49	44	31
**% Far Red (701–750 nm)**	6	5	2	3	2
**PPFD (μmol m**^**-2**^ **s**^**-1**^**)**	900	900	900	900	900
**YPF (μmol m**^**-2**^ **s**^**-1**^**)**	846	801	810	792	783
**Normalized YPF**	1	0.95	0.96	0.94	0.93
**Shortwave Radiation (280–4,000 nm; W m**^**-2**^**)**	327	222	230	235	253
	*332				
**Longwave Radiation (4,000–100,000 nm; W m**^**-2**^**)**	474	461	454	455	453
	*509				

Yield photon flux (YPF) was normalized to HPS. Clear-glass was mounted below the HPS to reduce longwave radiation.

*Radiation of HPS without glass mounted below the fixture. White+red 2 LED was switched with HPS without glass in trial three to quantify the effect of increased longwave radiation.

**Fig 2:**
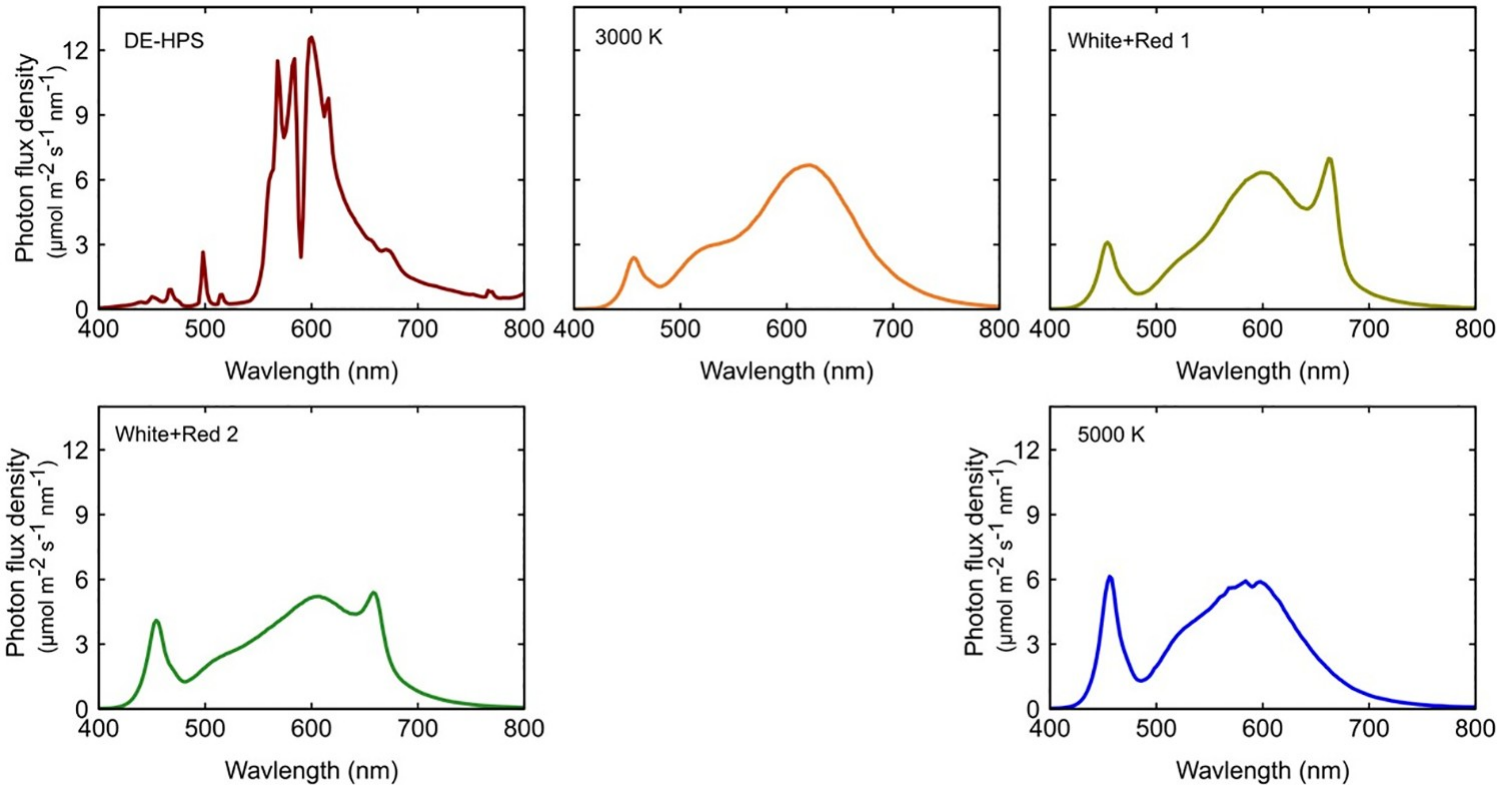
Spectral traces of a 1000 W DE-HPS and four LED fixtures. Measurements were made at a PPFD of 900 μmol m^-2^ s^-1^.

It is critical to maintain constant PPFD among spectral treatments within a study. PPFD at canopy height was kept constant throughout the study by raising the light fixtures as plants grew. PPFD measurements and fixture adjustments were made twice per week for the first four weeks of the flowering period, then once per week for the final four weeks. PPFD was measured using a full-spectrum quantum meter (Apogee Instruments Inc., model MQ-500). The highly reflective chamber walls and the fixture distance from the canopy minimized non-uniformity of lighting within a chamber. The PPFD in the corners of all chambers was within 15% of the average PPFD. Spectral traces of the five fixtures are shown in Fig 2. Measurements were made with a spectroradiometer (Apogee Instruments Inc., model PS-300). YPF was calculated using the weighting factors in Sager et al. (1988). The YPF (normalized to HPS) increased from 0.93 to 1.0 as the blue fraction decreased from 20% to 4% (Table 2).

HPS bulbs emit more longwave (thermal) radiation, causing higher leaf and flower bud temperature than LEDs at equal air temperature and PPFD [53]. In all studies, glass was mounted below an HPS fixture to make the longwave radiation similar to the LEDs (Table 2). Radiation measurements were made using a four-way net radiometer (Apogee Instruments Inc., model SN-500-SS). Canopy temperature was measured using a forward-looking infrared camera (FLIR Systems, model E6). In trial three, the white+red 2 LED fixture was replaced with an HPS without glass to quantify the effect of increased longwave radiation on canopy temperature and yield.

### Plant material

Rooted cuttings of the medical hemp cultivar ‘Trump’ (T1) were transplanted into #2 plastic pots (6.3 L) filled with a soilless mix of 3:1 peat/vermiculite that was amended with 1.6 g per liter of dolomitic lime to increase pH to 5.5. Gypsum (CaSO_4_ ·2H_2_O) was added at 0.8 g per liter to provide additional calcium and sulfur. The cultivar ‘Trump’ was selected because it has a compact growth habit and high cannabinoid concentrations. Plants were grown for 7 to 14 days (18 light:6 dark) and selected for uniformity before being switched to an inductive photoperiod (12 light:12 dark). Plant density was six plants per m^2^ in trials one and three and two plants per m^2^ in trial two. This facilitated testing a potential interaction of spectral quality with plant density and thus the rate of increase in photon capture.

Plants were irrigated daily to a 10% excess with a complete liquid fertilizer (Peter’s Peat-lite professional 20-10-20 [20N-4.4P-16.6K]) at a rate of 120 mg per liter N (26 mg/L P, 100 mg/L K, 1 mg/L Mg, 0.15 mg/L B, 0.15 mg/L Cu, 0.6 mg/L Fe, 0.3 mg/L Mn, 0.06 mg/L Mo, 0.3 mg/L Zn). Greencare micronutrients (Greencare Fertilizers, Inc.) were added at a rate of 7 mg per liter (0.12 mg/L B, 0.12 mg/L Cu, 0.49 mg/L Fe, 0.25 mg/L Mn, 0.05 mg/L Mo, 0.25 mg/L Zn). AgSil 16H (PQ Corporation) was added using a second proportioner for the liquid fertilizer at a rate of 8.4 mg/L Si (18 mg/L SiO_2_; 34 mg/L H_4_SiO_4_; 0.3 mmol Si per liter). Electrical conductivity (EC) and pH of the nutrient solution were 1.2 ± 0.1 mS cm^-1^ and 6.8 ± 0.1. Each pot had two 1 gallon-per-hour pressure compensating drip emitters (DIG, model B221B). Drip emitters were tested at the start and end of each trial to ensure uniformity.

### Plant measurements

Root zone status was monitored throughout the study by measuring EC and pH of leachate. Changes to irrigation duration or frequency were made to maintain a leachate EC of about 1.0 mS cm^-1^.

At approximately two weeks after the start of the inductive photoperiod and each week thereafter, flower samples were harvested from a subset of each spectral treatment to characterize the time course of cannabinoid accumulation. Flowers were sampled at a uniform height. Samples were air-dried for five days on well-ventilated shelves (25°C, 35% RH) to a moisture level of 10 ± 2% by weight.

At harvest, leaves, flowers, and stems were manually separated, weighed and dried. All fan leaves were separated from the flowers; the small (sugar) leaves that subtend the inflorescence were not separated from the flowers. After five days, dry mass was measured and a sample of the flowers from each plant was analyzed for final cannabinoid concentration. Trial two was terminated after seven weeks; trials one and three were terminated after eight weeks. The flower buds appeared physiologically mature at harvest in all trials.

### Cannabinoid extraction

Dried flower samples were ground to a coarse powder using a stainless-steel grinder (KitchenAid, model BCG111OB). A 0.6-gram subsample of the dried flower material was transferred to an aluminum extraction cell (Q-Cup) containing a 40 μm cellulose filter base (C9) topped with a 0.25 μm membrane filter (M2). Flower samples within the cells were extracted with reagent-grade methanol (10 mL top volume, 5 mL bottom volume, 5 mL top rinse; Fisher Scientific) using an Energized Dispersive Guided Extraction (EDGE^®^) automated solvent extraction system (CEM Co.) [54]. The temperature in the Q-Cup was maintained at 100°C with a hold time of 3 minutes. Samples were extracted twice to maximize recoveries. Blank cells were extracted between every sample.

### Cannabinoid analysis

Sample extracts were analyzed using an Agilent 1100 series (Agilent Technologies, Santa Clara, CA) reverse phase high-performance liquid chromatography (HPLC) equipped with diode array detector and a Kinetex 2.6 μm C18 polar 100 Å, 100 x 4.6 mm column (Phenomenex Inc.). The mobile phase was a 70/30 ratio of 0.1% formic acid-acetonitrile and 0.1% formic acid-distilled water and the flow rate was 1 mL per min. A reference standard containing the cannabinoid compounds of interest (Cayman Chemical Inc., Phytocannabinoid Qualitative Mixture 10) was used to prepare the external calibration standards. CBDA was analyzed at 210 nm and had a retention time of 2.2 min; CBD was analyzed at 230 nm and had a retention time of 2.9 min; THC was analyzed at 285 nm and had a retention time of 4.0 min; and THCA was analyzed at 275 nm and had a retention time of 4.6 min. Minor cannabinoids such as CBG were excluded from the analysis due to low concentrations. Peaks were auto-integrated using ChemStation (Agilent Technologies). CBD and THC equivalents (CBD_eq_ and THC_eq_) are calculated using Eqs 1 and 2. These account for the conversion of the naturally produced acidic cannabinoids to the neutral form (decarboxylation). The 0.877 multiplier is the molecular weight ratio of the neutral form to the acidic form [55].

CBDeq=CBD+(CBDA*0.877) (1)

THCeq=THC+(THCA*0.877) (2)

### Statistical analysis

The study was a randomized block design with the three studies in time as blocks. Environmental conditions and plant density were changed among studies to determine potential interactions of the blue photon treatment with other factors. This approach is similar to three replicate studies in the field over three growing seasons. To eliminate a potential chamber effect, treatments were randomly assigned to a chamber between trials. Each study included five fixed blue photon fraction levels. The community of plants in each chamber (bordered by the reflective walls) was treated as an experimental unit, rather than treating individual plants within a chamber as replicates. Yield was calculated as the total mass of dry flower per unit area.

The data were analyzed with a linear mixed model using the LME4 package in RStudio (R statistical software, version 1.2). Blue photon fraction was treated as a fixed effect and trial was treated as a random effect. One spectral treatment (white+red2) was exchanged in trial three to provide an HPS without glass treatment. This meant that HPS with glass and three LED treatments had three replicates in time, the white+red2 had two replicates in time, and the HPS without glass was not replicated. The estimated trial variance on slope (blue photon fraction) was zero, which indicates a common slope for the three trials. Therefore, a random intercept regression model was used for further analysis in which intercept varied due to trials with a fixed blue photon fraction slope for all trials [56].

## Results

### Yield

**Fig 3:**
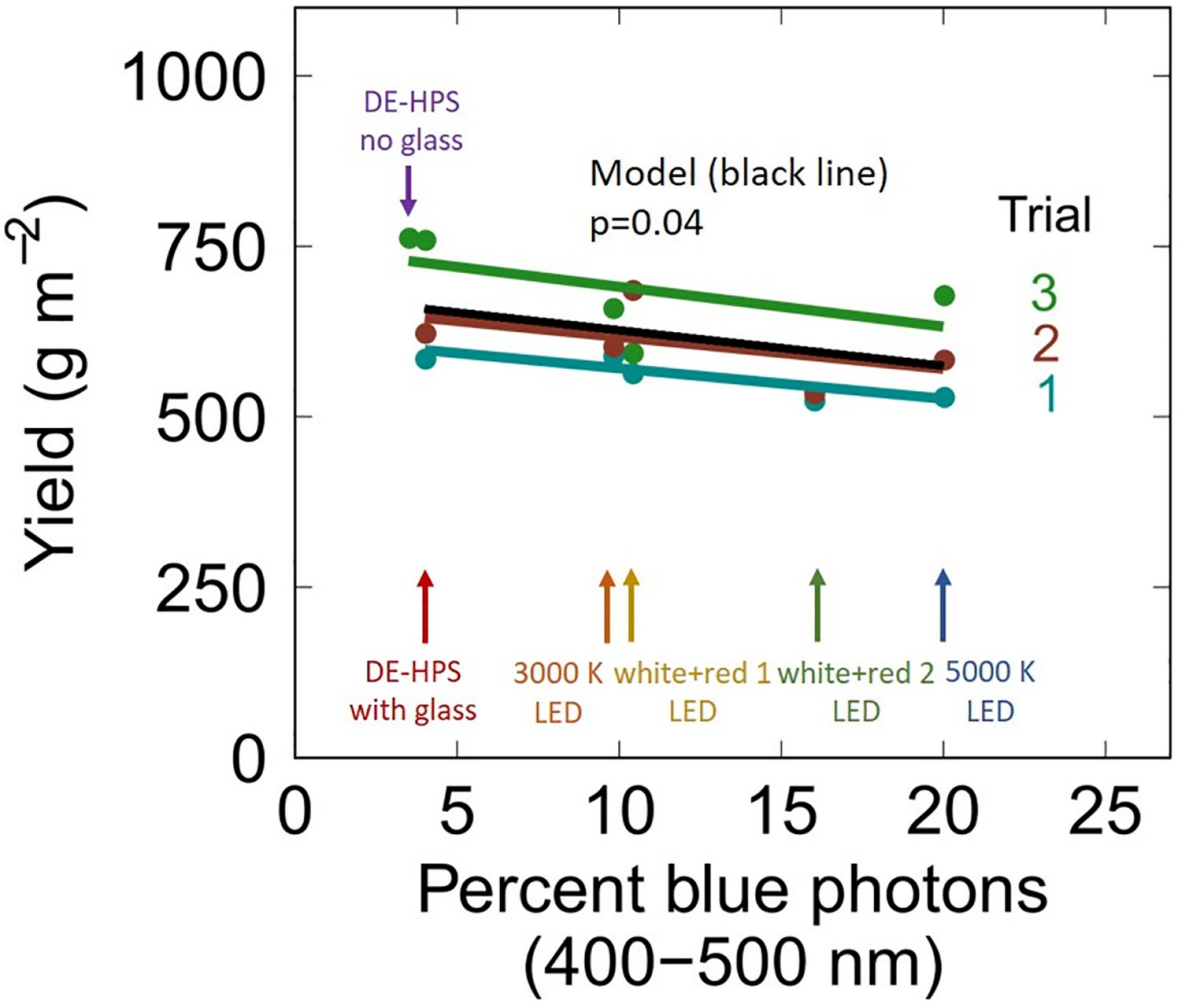
The effect of blue photons on flower yield in three trials. Fixture names of each blue photon fraction are shown with arrows. The black line represents the linear mixed model with blue photon fraction as a fixed effect and trial as a random effect. The slopes of the lines for the three studies were parallel, indicating no interaction among studies. Yield increased by 0.77% per 1% decrease in blue photons (p = 0.04).

As percent blue increased from 4 to 20%, flower yield decreased by 12.3%. This means that flower yield increased by 0.77% per 1% decrease in blue photons (Fig 3; p = 0.04). It is important to note that there was no significant interaction of blue photon fraction with trial (indicated by parallel slopes among the three trials). This indicates a consistent effect of percent blue across a range of yields and environments (Fig 3).

Harvest index (HI) is the ratio of usable biomass to total biomass, here defined as the ratio of dried flower to total dry above-ground mass (flowers, leaves and stems). Typical values for HI in crop plants range from 30 to 50% [57]. HI ranged from 55 to 65% among spectral treatments in the three trials, but there was no effect of blue photons on HI (S1 Fig; p = 0.91). There was no significant interaction with trial on harvest index.

The effect of blue photon fraction on height was not statistically significant (data not shown; p = 0.13).

### Cannabinoid concentration

**Fig 4:**
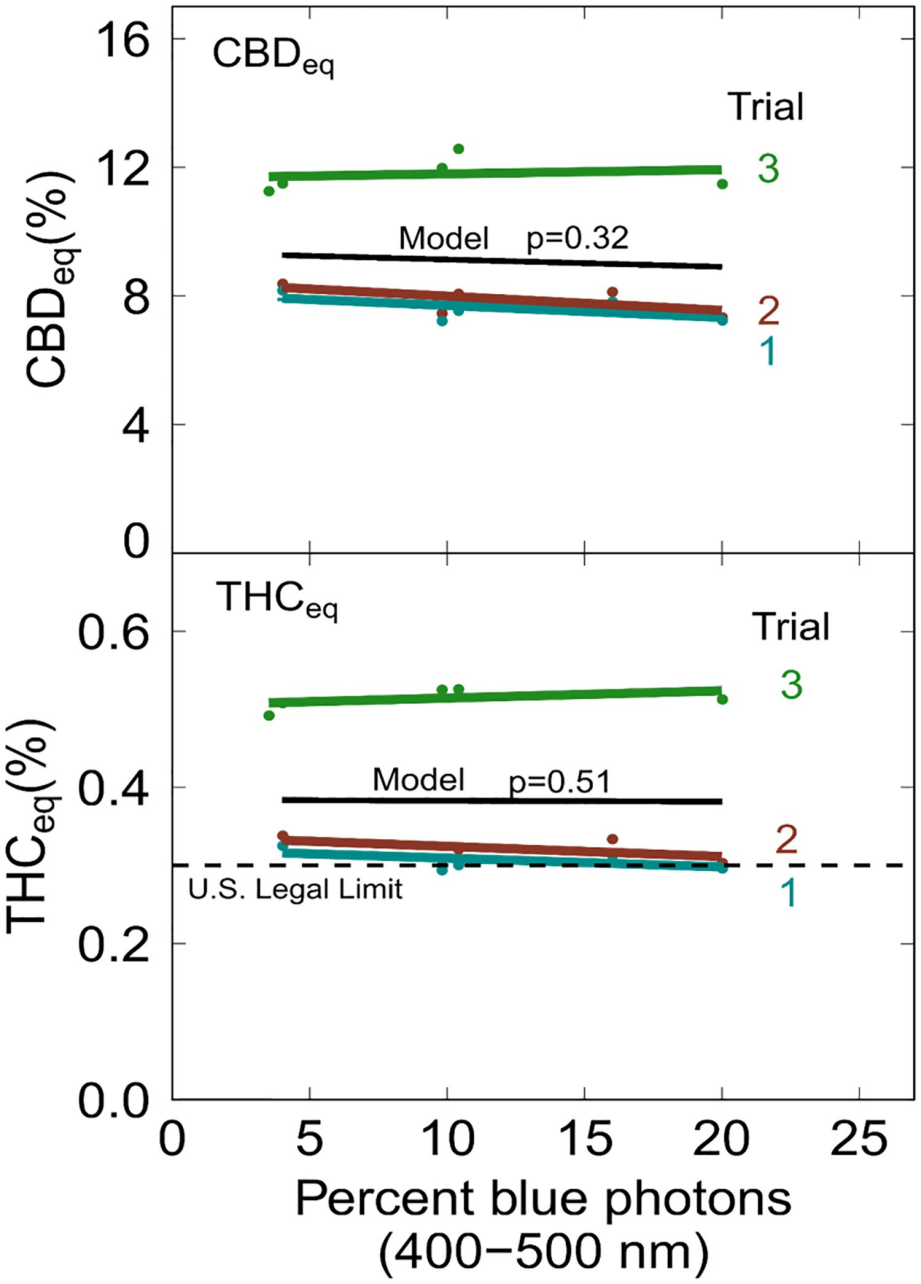
The effect of blue photons on (A) CBD_eq_ and (B) THC_eq_ concentration at harvest. The black line represents the linear mixed model with percent blue photons as a fixed effect and trial as a random effect. There was no significant effect of blue photon fraction on CBD_eq_ (p = 0.32) or THC_eq_ (p = 0.51) concentration at harvest.

Blue photon fraction had no effect on final cannabinoid concentration (Fig 4A and 4B; CBD_eq_ p = 0.32; THC_eq_ p = 0.51).

**Fig 5:**
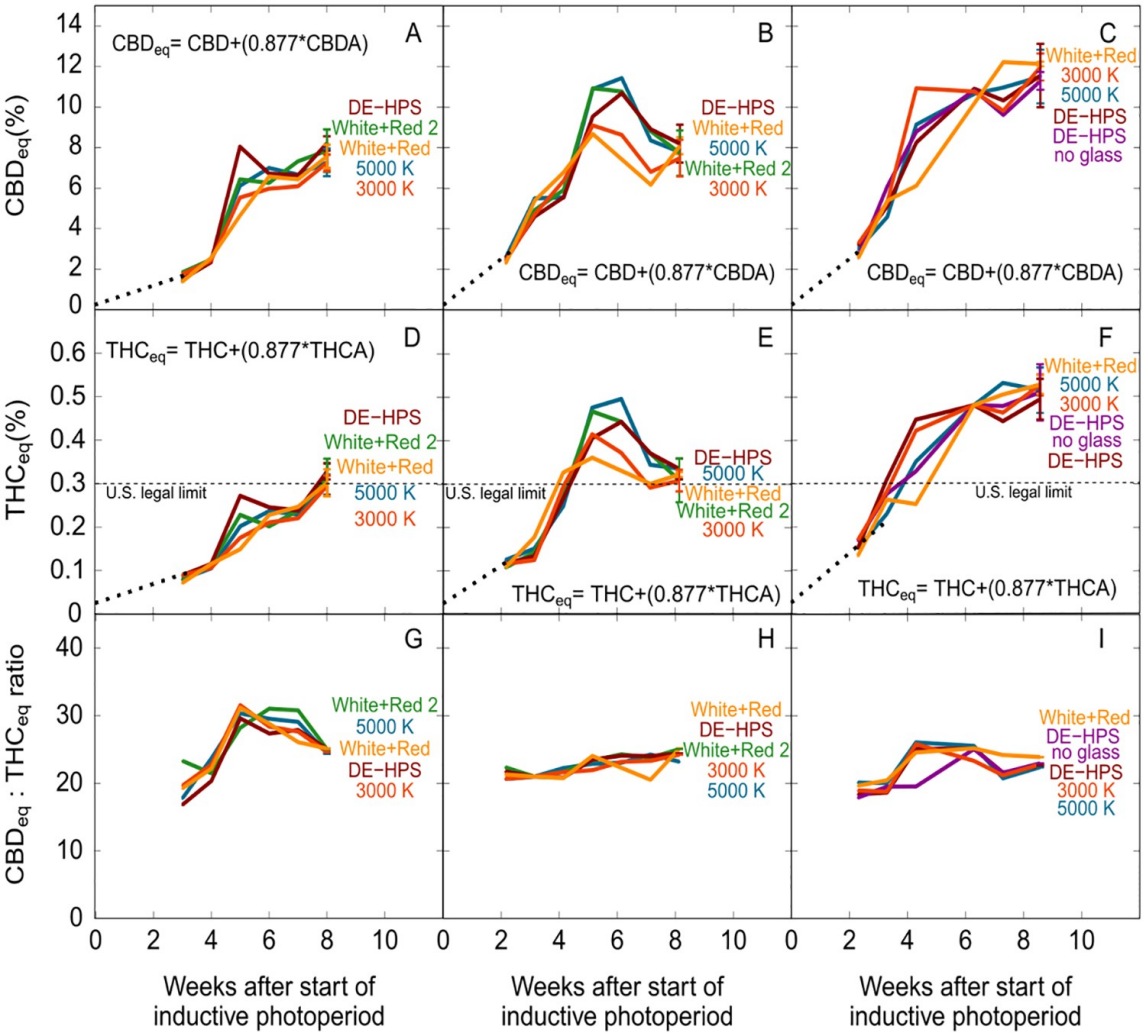
Time course of (A-C) CBD_eq_ and (D-E) THC_eq_, accumulation, and (G-I) CBD_eq_ to THC_eq_ ratio over the flowering period among five spectral treatments. All points, until final harvest, are single samples. Multiple replicate samples were analyzed at final harvest as shown by the standard deviation error bars.

In trial one, the ratio of CBD_eq_ to THC_eq_ rose to 34 to 1, but dropped to 25 to 1 at harvest (Fig 5G). In trials two and three the ratio was approximately 25 to 1 at each sampling point (Fig 5H and 5I). The final ratio of CBD_eq_ to THC_eq_ was 24 to 1 in all treatments and trials. In trial one, the THC_eq_ concentration was 0.31 ± 0.03% at harvest while in trial three, the THC_eq_ concentration increased to 0.52 ± 0.06%. In trial two, the concentration of THC_eq_ rose to 0.46 ± 0.07% at about week five, but then dropped to 0.32 ± 0.03% at harvest (Fig 5D–5F).

In trial one, the concentration of CBD_eq_ was about 7.6 ± 0.7% at harvest, while in trial three the concentration of CBD_eq_ increased to 11.8 ± 1%. In trial two, the concentration of CBD_eq_ rose to 11 ± 1.7% at week 5, but then dropped to 7.9 ± 0.7% at harvest (Fig 5A–5C).

### Efficacy

An advantage of LED fixtures is their high efficacy and thus low electrical (operating) costs compared to HPS. At an energy cost of $0.10 USD per kWh, using the efficacies listed in Table 2, it costs 1.6 cents per mol of photons from an HPS fixture and 1.1 to 1.3 cents per mol for the LEDs (S2 Fig).

**Fig 6:**
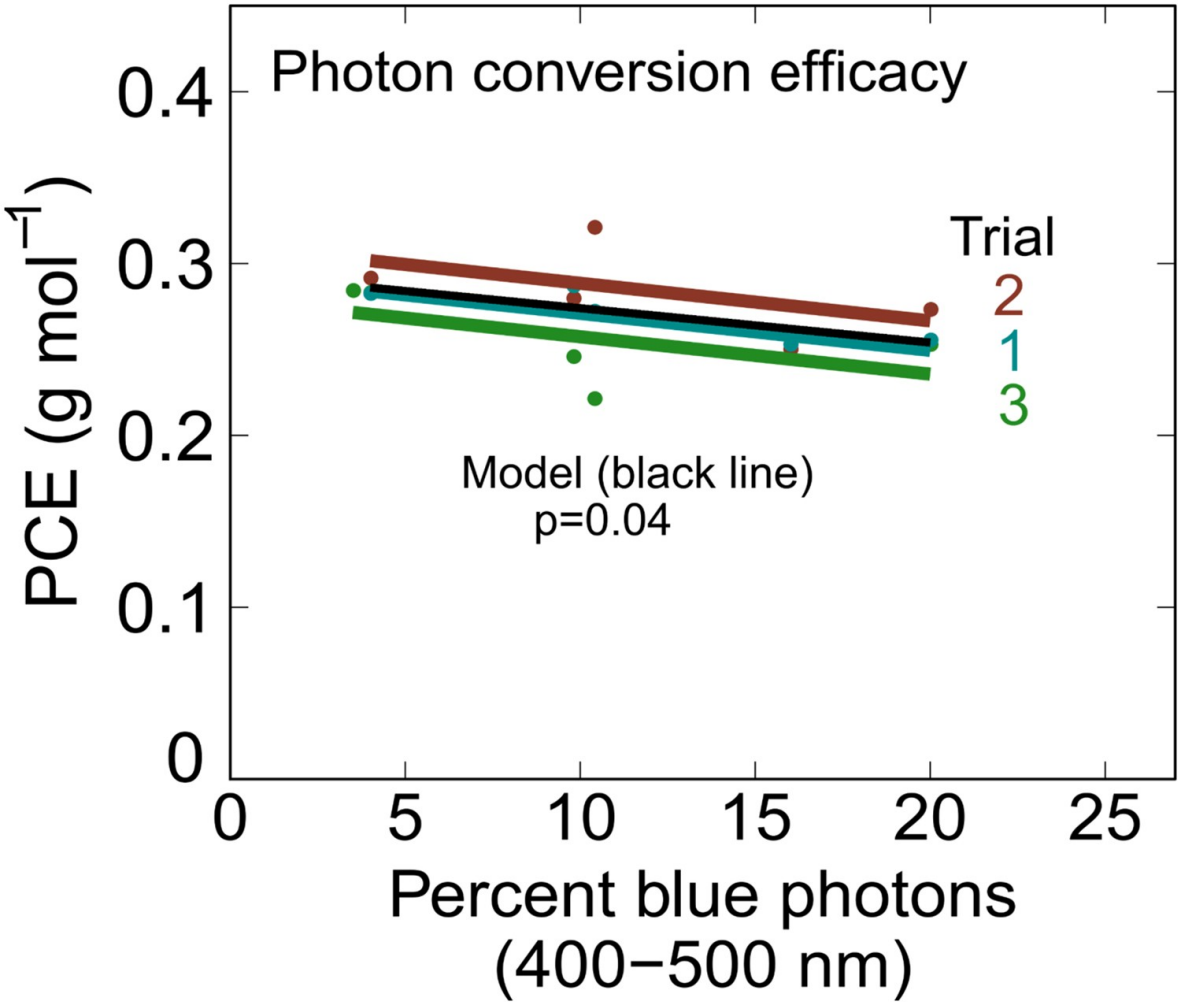
Effect of blue photons on photon conversion efficacy (PCE). PCE increased with decreasing blue photon fraction (p = 0.04).

Photon conversion efficacy (PCE) is calculated by dividing dry flower yield (g m^-2^ d^-1^) by the daily light integral (DLI; mol m^-2^ d^-1^). PCE ranged from 0.22 to 0.36 g per mol, based on photon integration from transplanting to harvest (Fig 6). It is important to include the photons during the 7 to 14-day vegetative growth phase in this analysis. In these studies, the PCE would have been about 10% higher with closer pot spacing during the vegetative growth phase. The canopy closed (filled the entire chamber) in all trials by about week 3. PCE is calculated per unit ground area, not per plant area. A lower plant density would thus typically reduce photon capture and PCE.

**Fig 7:**
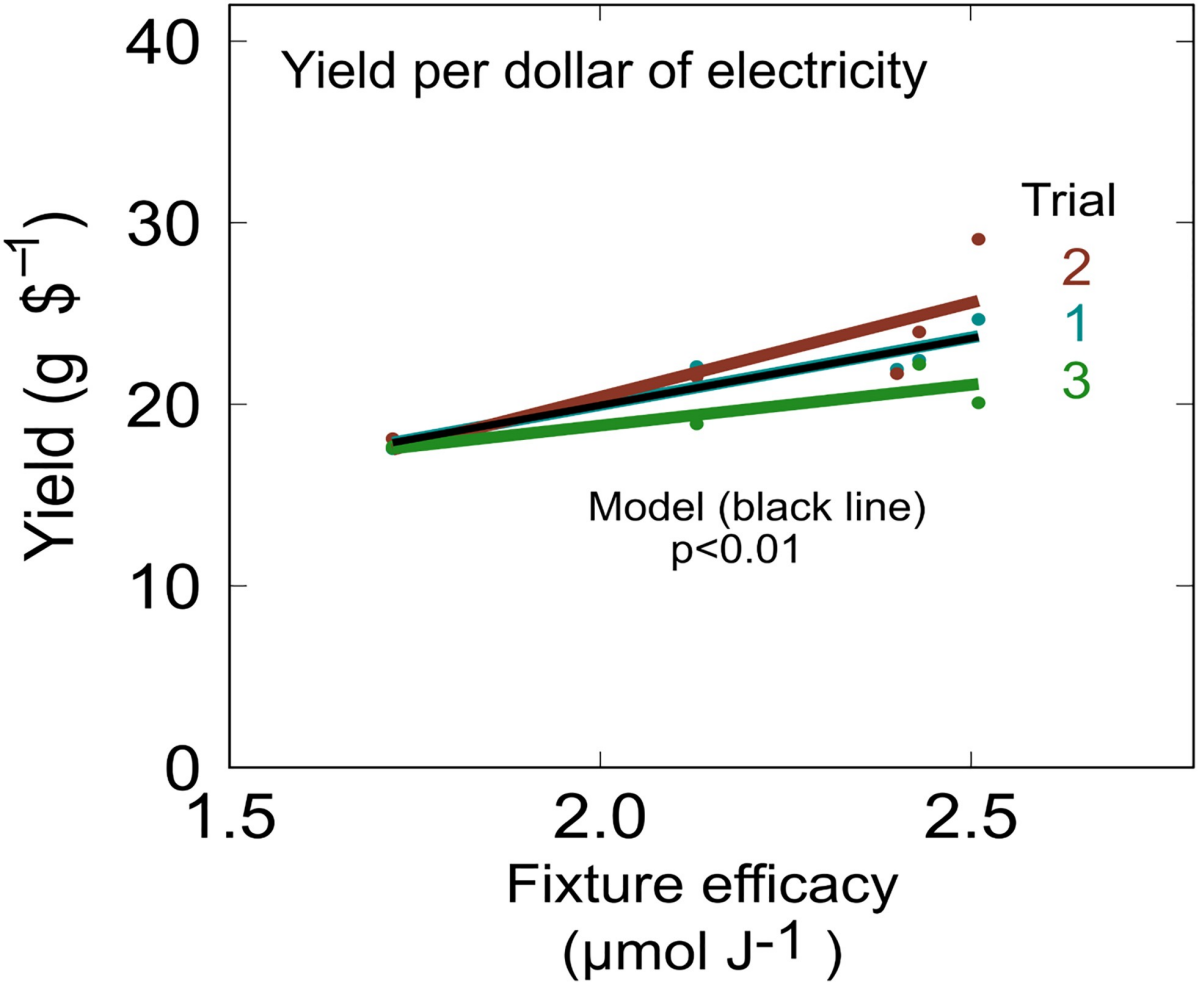
Effect of fixture efficacy on yield per dollar of electricity. Assumes an energy cost of $0.10 per kWh. Yield per dollar of electricity significantly increased as fixture efficacy increased (p<0.01).

PCE values facilitate calculation of flower yield per dollar of electrical energy for the light fixtures. Yield per dollar of electricity increased as efficacy increased in all trials (Fig 7; p<0.01). The white+red 1 fixture (10% blue) had the highest efficacy (2.51 μmol J^-1^) and produced 27% more flower per dollar of electricity on average than HPS and 16% more flower per dollar than the other LEDs with lower efficacy.

## Discussion

### Yield

#### Potential underlying physiological basis for the observed yield reduction

Analyzing the results by YPF indicates that a decrease in quantum yield with an increasing blue photon fraction would account for 7% of the 12% decrease in yield (S6 Fig). Although leaf area was not measured, photon capture may have also contributed to the yield reduction. Far-red photons likely had a small contribution to the 12% decrease in yield. Thus, four physiological responses could have contributed to the 12% decrease in yield: 1) blue fraction effect on quantum yield, 2) blue fraction effect on leaf expansion and photon capture, 3) far-red fraction effect on photosynthesis, and 4) far-red fraction effect on leaf expansion and photon capture.

1. Blue photons have a lower quantum yield due to photon absorbance by non-photosynthetic pigments within leaves [9].
2. Increasing the fraction of blue photons is typically associated with decreased leaf expansion and thus reduced photon capture [17, 19].
3. Far-red photons from 701 to 750 nm are photosynthetically active [14, 15].
4. Far-red photons can increase yield by modifying morphology and increasing photon capture [25].

There is the potential that changes in the fraction of other wavelengths could have contributed to the results. Percent blue ranged from 4 to 20% (a 5-fold increase), percent green ranged from 39 to 49%, percent red ranged from 31 to 53%, and percent far-red ranged from 2 to 6%. Snowden et al. [19] found that changes in green photon fraction from 2 to 41% had a minimal effect of growth of seven species, so the 6% range in these studies likely had a minimal effect on yield or yield parameters. The shift from blue to red photons likely increased photosynthesis per incident photon, but considering the five-fold change in blue photon fraction, and the associated effects on morphology, it is unlikely that the 22% change in red photon fraction was the primary factor underlying the growth and yield effects.

For the above reasons, it is unlikely that the changes in green or red photon fraction were the primary factors responsible for the effects on yield. This is consistent with previous studies on other species [17–19].

These results are similar to those of Magagnini et al. [6] who reported a decrease in flower yield in *Cannabis* (in one of two replicate studies) when the fraction of blue increased from 8 to 14%, but there was no further change from increasing the fraction of blue from 14 to 24%. We found a linear decrease in flower yield up to 20% blue photons. The PPFD in this study was double the intensity of Magagnini et al. [6], which may have affected the response.

#### Effects of other environmental factors

There were differences in average daily temperature and day night differential among studies, *but the effect of blue photons was consistent in all studies*. The lower temperature and lower PPFD in trial one likely contributed to the lower yield compared to trials two and three.

The canopy temperature in the unshielded HPS treatment averaged 0.3°C higher than the shielded HPS, and 0.9°C higher than the LED treatments (S3 Fig). This is consistent with the leaf and canopy temperature model of Nelson and Bugbee [53]. Although the unshielded HPS treatment was only replicated in one study, yield was within 3% of the yield of the other three replicate HPS treatments.

The photosynthetic response of plants to PPFD is non-linear, so the effect of intensity becomes less significant at high PPFD [26]. The photosynthetic response of plants to temperature, especially between 20° and 30°C, is generally more significant and is expected to have a larger effect on photosynthesis [58]. The optimum temperature for photosynthesis is variable among species and is typically related to the region of origin of a species [59]. Bazzaz et al. [60] determined that *Cannabis* acclimated to warmer temperatures (32°C/23°C, day/night) had a higher photosynthetic rate than plants acclimated to cooler temperatures (23°C/16°C, day/night). The difference in yield among trials is therefore likely due to differences in temperature, but further research is needed to understand the effect of temperature on flower yield of *Cannabis*. In any case, the effect of spectra on yield was consistent among trials.

It is possible that *Cannabis* cultivars with unique morphologies and days to flower would respond differently to blue photon fraction. The effects of blue photon fraction vary among species [19]. Although growth parameters (e.g. height, fresh weight) vary among cultivars within a species, the effects of spectra on these parameters are expected to be small [23]. About 7% of the 12% reduction in yield can be accounted for by lower YPF, a response that is consistent across species [9]. The cultivar “Trump” was selected from among 12 diverse cultivars because it represents an average time to flower. Future research is needed to evaluate potential interactions of spectra with *Cannabis* cultivars.

### Cannabinoid concentration

#### Spectral effects

Spectral quality can trigger synthesis of secondary metabolites, which include photo-protective pigments (e.g. anthocyanins), but no theoretical mechanism that links spectral quality to cannabinoid biosynthesis has been elucidated. In this study, there was no statistical difference in final cannabinoid concentration among spectral treatments. Magagnini et al. [6] reported an increase in CBD and THC concentration under 14 and 24% blue (from LEDs) compared to 8% blue under a mogul-base HPS fixture. Additionally, they saw an increase in CBG concentration, a precursor to both CBD and THC, with an increasing fraction of blue photons. They hypothesized that the first enzyme in the cannabinoid pathway is responsive to blue photons. Photoreceptors are likely under-saturated at lower light intensities allowing for an increased sensitivity to spectral quality. This could explain why an effect on cannabinoid concentration was observed at the lower PPFD of Magagnini et al. [6] and not at the higher PPFD in this study.

#### Time-course of cannabinoid accumulation

Cannabinoid biosynthesis is relatively well understood, although the effects of environment are not well characterized. Cannabinoid degradation, especially *in vivo*, is far less studied but the implications could be significant. In trial two, both CBD and THC peaked around week five followed by a decrease in the last two weeks of flowering. This decrease has been reported previously [61, 62]. It is not necessarily caused by temperature. The underlying effect of environment on cannabinoid accumulation is not yet clear.

Growth dilution could explain a decline in the final weeks of flowering. This occurs when the plant accumulates biomass faster than cannabinoids. Alternatively, cannabinoids could be degrading. Mahlberg and Kim [34] indicate that trichome maturity can be assessed by color, which is related to the relative cannabinoid content. They found that mature glands are translucent and contain the highest cannabinoid concentration, aged glands are yellow and contain lower cannabinoids and senescent glands are black or brown and contain the lowest cannabinoids. They propose this could be caused by polymerization, transportation, or volatilization of cannabinoids but the mechanism remains to be elucidated. Regardless of the mechanism, the fact that THC concentration can decline in the final weeks of flowering has implications for hemp growers where regulations dictate a maximum THC concentration. Further research is needed to determine the stability and longevity of cannabinoids *in vivo*, and whether environmental factors can be altered to reduce THC.

### Fixture design and efficacy

LEDs have facilitated rapid progress in understanding spectral effects on plant growth and secondary metabolism, but these data indicate that efficacy has a larger effect than spectra on the economics of indoor *Cannabis* cultivation. Decreasing the blue fraction from 20% to 4% resulted in a 12.3% increase in dry mass yield, while increasing the efficacy from 1.72 to 2.51 μmol J^-1^ resulted in a 27% increase in yield per dollar of electricity. Similar results were reported for 13 lettuce cultivars grown in greenhouses supplemented with HPS or LED lights [23].

Red LEDs have a higher efficacy than blue (and by proxy white) LEDs because red photons have less energy than green and blue. This indicates that LED fixture manufacturers and growers should consider white+red fixtures that have a high portion of red [5]. The white+red 1 (10% blue) treatment had the highest yield per dollar of electricity. The highest efficacy fixtures listed by the Design Lights Consortium are white+red or blue+red combinations [52].

One drawback of high fraction red LED fixtures is low color rendering index (CRI; R_a_). This metric indicates how well a light source reveals the colors of an object relative to a reference, usually an incandescent light or sunlight. A newer metric, color fidelity index (CFI; R_f_), which comes from Technical Memo 30 (TM-30), is mathematically more complex and is designed to provide an improved metric to assess the unique mixtures of colors in LED fixtures [63]. Both CRI and CFI range from 0 to 100. These metrics are routinely used for human lighting applications but are rarely considered in controlled environment agriculture. Broad-spectrum light sources (usually with white LEDs) have a higher CRI and CFI than narrow-spectrum sources [64]. Unfortunately, high-efficacy green LEDs are not yet available, but green photon fraction increases the CRI and CFI. This facilitates identification of pests, pathogens, or nutrient disorders. HPS has a CRI of 43 and a CFI of 55. White LEDs typically have a CRI above 70 and a CFI above 85 [65].

A photograph of plants under a DE-HPS and a 3000 K LED is shown in S4 Fig. There is a tradeoff between CRI/CFI and efficacy (S5 Fig). The importance of establishing a baseline CFI that optimizes identification of abnormalities while still maximizing efficacy is an overlooked parameter in horticultural lighting.

Optimized lighting is critical to the economics of indoor *Cannabis* cultivation. These data indicate that efficacy is more important than spectra, and that fixture manufacturers should consider reducing the blue fraction to improve economic yield of Cannabis.

## Supporting information

S1 Fig Effect of blue photons on harvest index (HI) in three studies. HI is the ratio of useable biomass to total above ground biomass, here defined as the ratio of flowers to flowers, leaves and stems. There was no significant effect of blue photons on HI (p = 0.91). HI was highest in trial one, which had the highest average temperature of the three trials.
(TIF)

S2 Fig Example calculation to determine grams per dollar of electricity. PPFD, efficacy, photon conversion efficiency (g per mol), and cost of electricity are inputs to the calculation. Assuming $0.10 per kWh and 0.3 g per mol, it costs $1.00 to produce 3 g of flower.
(TIF)

S3 Fig Thermal images of the canopy under five spectral treatments from trial three. Darker shades indicate cooler temperatures. Note that the flower buds (white color) are about 2 C warmer than the leaves. HPS without glass was about 1 C warmer than the LEDs and about 0.3 C warmer than the HPS with glass. Thermal images can be used to detect water stress or disease before visual symptoms become apparent.
(TIF)

S4 Fig Photograph of two adjacent spectral treatments separated by the chamber wall. The color rendering index (CRI) and color fidelity index (CFI) is low under HPS lights. White LEDs allow for easier identification of pests, pathogens, or nutrient disorders.
(TIF)

S5 Fig Effect of blue photon fraction on efficacy and CRI (top graph) and CFI (bottom graph) of an HPS fixture and four LED fixtures. CRI and CFI describe the ability of a light source to distinguish true colors of an object relative to a reference. Fixtures with a higher CRI or CFI typically have lower efficacy. HPS has low CRI, CFI and efficacy compared to LEDs.
(TIF)

S6 Fig Effect of blue photon fraction on canopy photosynthesis. Canopy photosynthesis measurements were made under blue+red LEDs. The system used to make measurements has been described previously [14, 15], and references cited therein]. Decreasing the blue photon fraction significantly increased canopy photosynthesis (p = 0.01).
(TIF)

S1 Data (CSV)
