# Supplementary figures and images for: Cannabis lighting: Decreasing blue photon fraction increases yield but efficacy is more important for cost effective production of cannabinoids
Source: PLoS One. 2021 Mar 23;16(3):e0248988. doi: 10.1371/journal.pone.0248988 (PMC7987162; doi:10.1371/journal.pone.0248988)

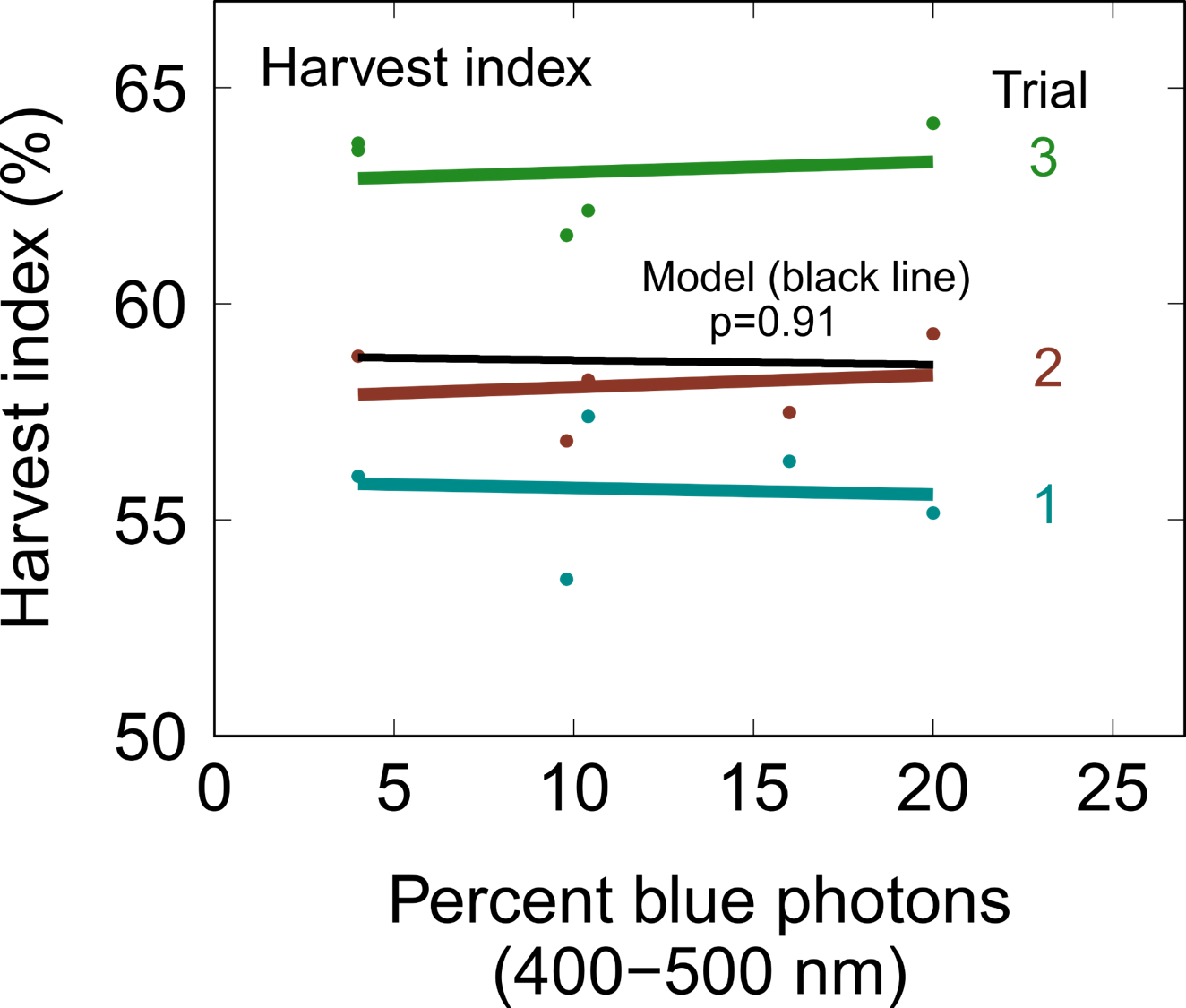

Supplement: S1 Fig — HI is the ratio of useable biomass to total above ground biomass, here defined as the ratio of flowers to flowers, leaves and stems. There was no significant effect of blue photons on HI (p = 0.91). HI was highest in trial one, which had the highest average temperature of the three trials. (TIF) [file pone.0248988.s001.tif]

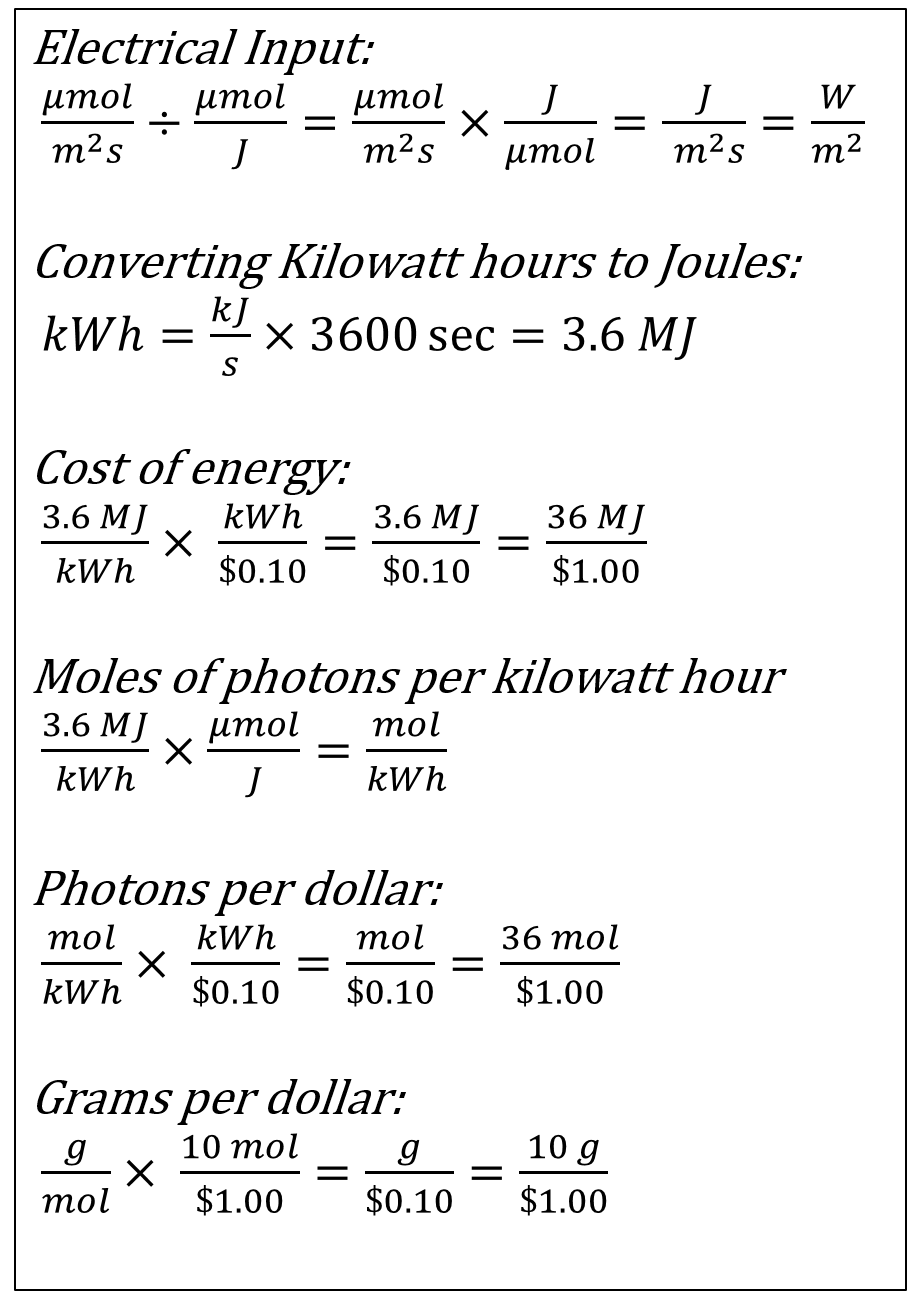

Supplement: S2 Fig — PPFD, efficacy, photon conversion efficiency (g per mol), and cost of electricity are inputs to the calculation. Assuming $0.10 per kWh and 0.3 g per mol, it costs $1.00 to produce 3 g of flower. (TIF) [file pone.0248988.s002.tif]

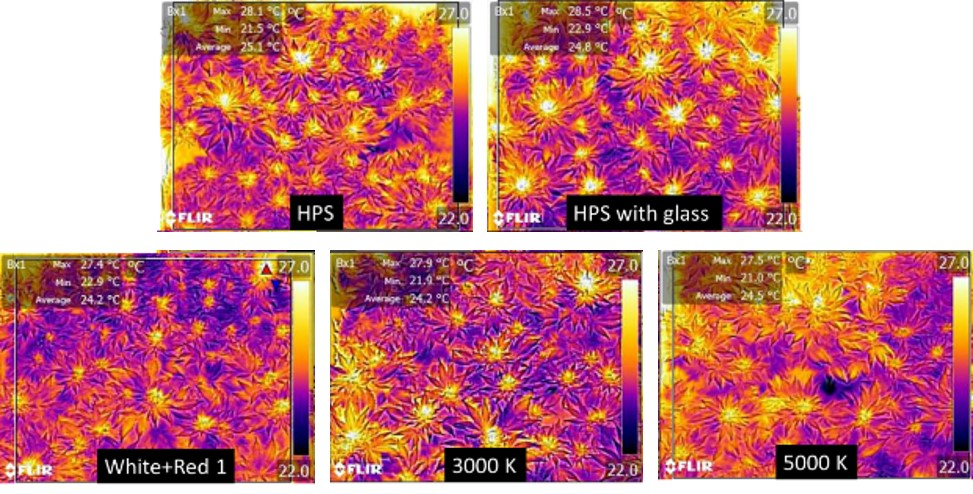

Supplement: S3 Fig — Darker shades indicate cooler temperatures. Note that the flower buds (white color) are about 2 C warmer than the leaves. HPS without glass was about 1 C warmer than the LEDs and about 0.3 C warmer than the HPS with glass. Thermal images can be used to detect water stress or disease before visual symptoms become apparent. (TIF) [file pone.0248988.s003.tif]

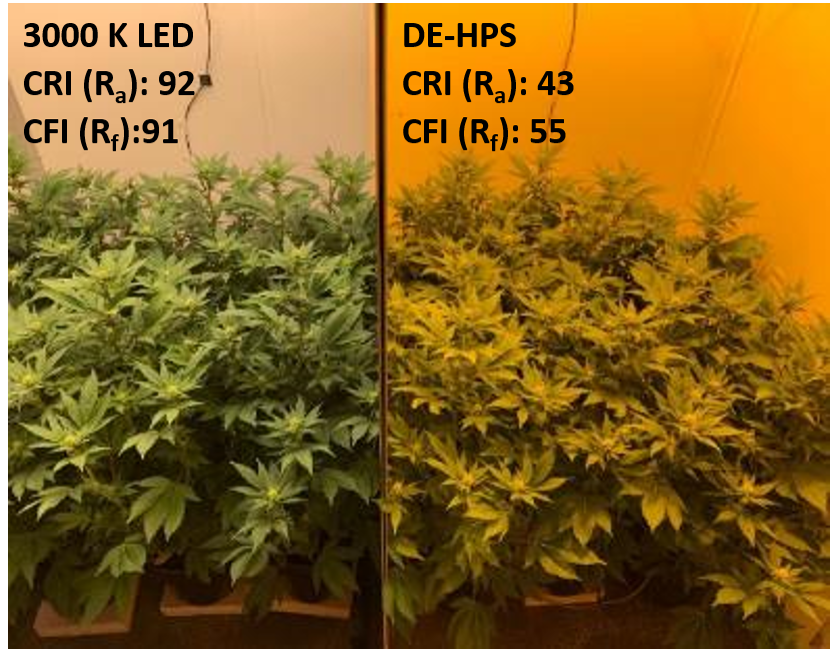

Supplement: S4 Fig — The color rendering index (CRI) and color fidelity index (CFI) is low under HPS lights. White LEDs allow for easier identification of pests, pathogens, or nutrient disorders. (TIF) [file pone.0248988.s004.tif]

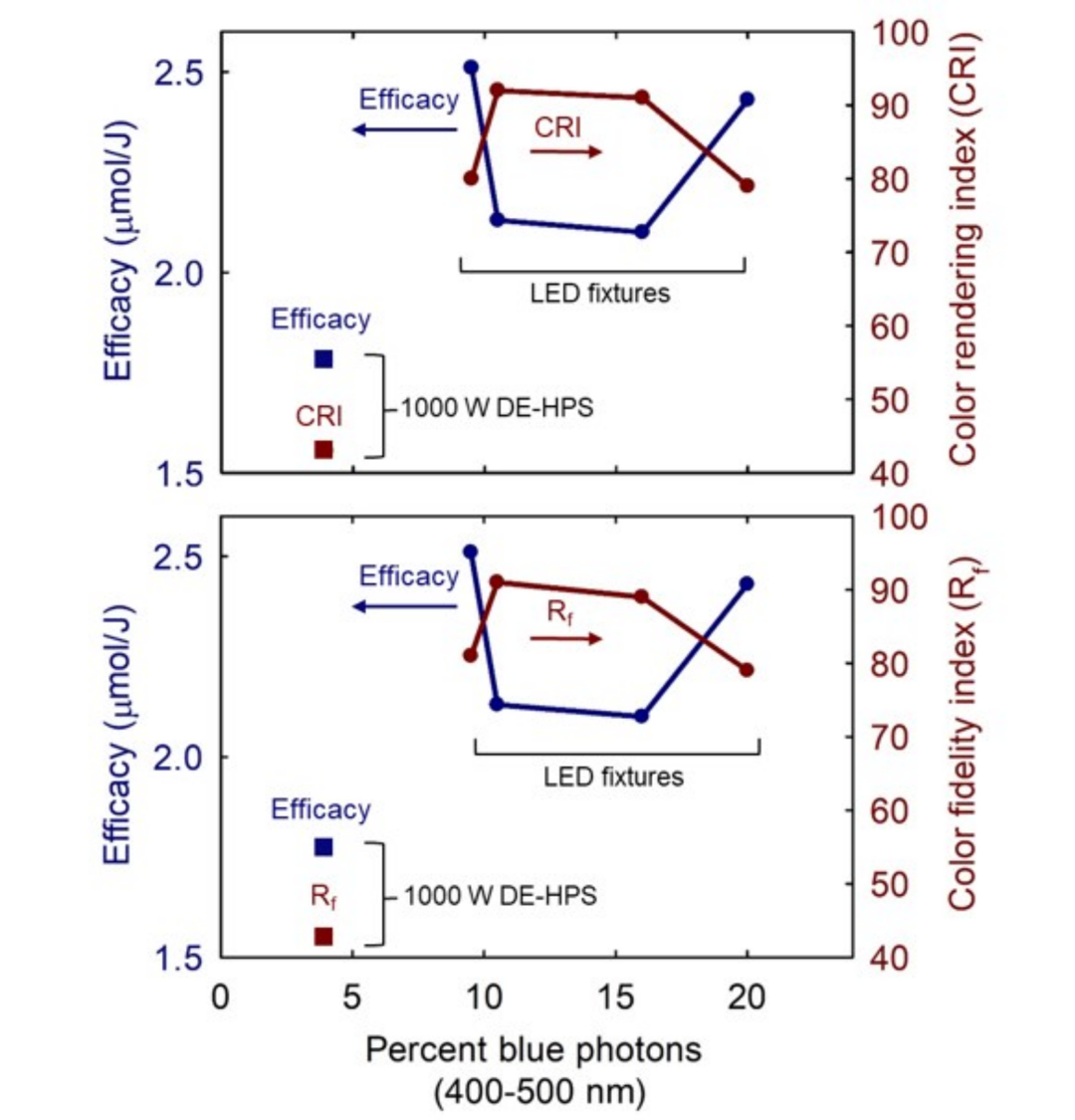

Supplement: S5 Fig — CRI and CFI describe the ability of a light source to distinguish true colors of an object relative to a reference. Fixtures with a higher CRI or CFI typically have lower efficacy. HPS has low CRI, CFI and efficacy compared to LEDs. (TIF) [file pone.0248988.s005.tif]

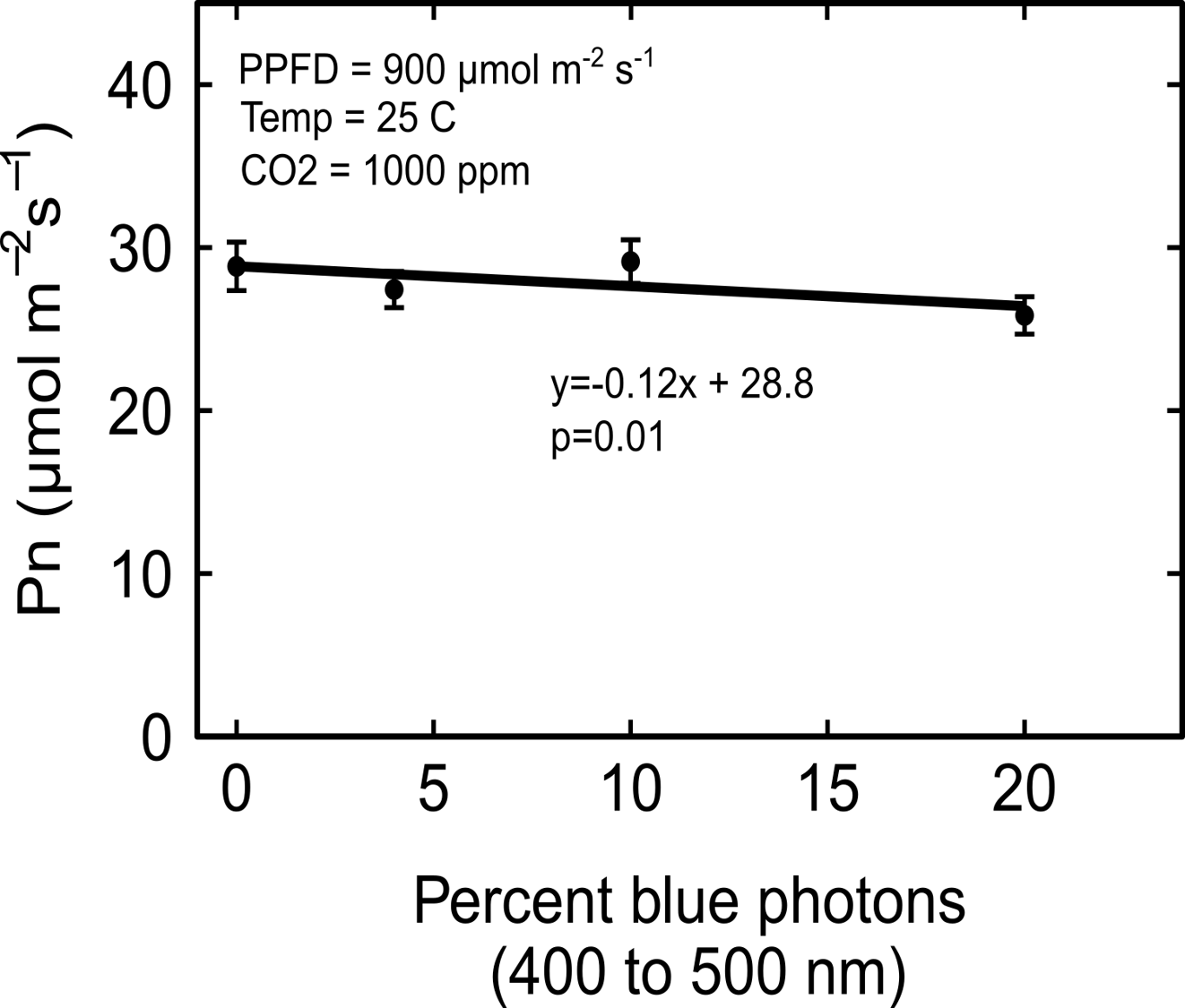

Supplement: S6 Fig — Canopy photosynthesis measurements were made under blue+red LEDs. The system used to make measurements has been described previously [14, 15], and references cited therein]. Decreasing the blue photon fraction significantly increased canopy photosynthesis (p = 0.01). (TIF) [file pone.0248988.s006.tif]
